# One-year postpartum anthropometric outcomes in mothers and children in the LIFE-Moms lifestyle intervention clinical trials

**DOI:** 10.1038/s41366-019-0410-4

**Published:** 2019-07-10

**Authors:** Suzanne Phelan, Rebecca G. Clifton, Debra Haire-Joshu, Leanne M. Redman, Linda Van Horn, Mary Evans, Kaumudi Joshipura, Kimberly A. Couch, S. Sonia Arteaga, Alison G. Cahill, Kimberly L. Drews, Paul W. Franks, Dympna Gallagher, Jami L. Josefson, Samuel Klein, William C. Knowler, Corby K. Martin, Alan M. Peaceman, Elizabeth A. Thom, Rena R. Wing, Susan Z. Yanovski, Xavier Pi-Sunyer, The LIFE-Moms Research Group

**Affiliations:** 1 Department of Kinesiology & Public Health, California Polytechnic State University, San Luis Obispo, CA USA; 2 The Biostatistics Center, George Washington University, Washington, DC USA; 3 Center for Diabetes Translation Research, Washington University in St. Louis, St. Louis, MO USA; 4 Pennington Biomedical Research Center, Baton Rouge, LA USA; 5 Department of Preventive Medicine, Northwestern University, Feinberg School of Medicine, Chicago, IL USA; 6 The National Institute of Diabetes and Digestive and Kidney Diseases, Bethesda, MD USA; 7 Center for Clinical Research and Health Promotion, School of Dental Medicine, Medical Sciences Campus, University of Puerto Rico, San Juan, Puerto Rico; 8 Department of Epidemiology, Harvard T.H. Chan School of Public Health, Harvard University, Boston, MA USA; 9 Phoenix Indian Medical Center, Indian Health Service, Phoenix, AZ USA; 10 The National Heart, Lung, and Blood Institute, Bethesda, MD USA; 11 Department of Obstetrics and Gynecology, Washington University in St. Louis, St. Louis, MO USA; 12 Department of Nutrition, Harvard T.H. Chan Public Health School, Harvard University, Boston, MA USA; 13 New York Obesity Research Center, Dept. of Medicine, College of Physicians and Surgeons, Columbia University, New York, NY USA; 14 Institute of Human Nutrition, College of Physicians and Surgeons, Columbia University, New York, NY USA; 15 Department of Pediatrics, Northwestern University, Feinberg School of Medicine, Chicago, IL USA; 16 Department of Clinical Sciences, Genetic and Molecular Epidemiology Unit Lund University Diabetes Centre, Skåne University Hospital Malmö, Malmö, Sweden; 17 Center for Human Nutrition, Washington University in St. Louis, St. Louis, MO USA; 18 Diabetes Epidemiology and Clinical Research Section, National Institute of Diabetes and Digestive and Kidney Diseases, Phoenix, AZ USA; 19 Department of Obstetrics and Gynecology, Northwestern University, Feinberg School of Medicine, Chicago, IL USA; 20 The Miriam Hospital and the Department of Psychiatry and Human Behavior, Warren Alpert Medical School at Brown University, Providence, RI USA

**Keywords:** Weight management, Paediatrics

## Abstract

**Background/objectives:**

Excess gestational weight gain (GWG) is a risk factor for maternal postpartum weight retention and excessive neonatal adiposity, especially in women with overweight or obesity. Whether lifestyle interventions to reduce excess GWG also reduce 12-month maternal postpartum weight retention and infant weight-for-length *z* score is unknown. Randomized controlled trials from the LIFE-Moms consortium investigated lifestyle interventions that began in pregnancy and tested whether there was benefit through 12 months on maternal postpartum weight retention (i.e., the difference in weight from early pregnancy to 12 months) and infant-weight-for-length *z* scores.

**Subjects/methods:**

In LIFE-Moms, women (*N* = 1150; 14.1 weeks gestation at enrollment) with overweight or obesity were randomized within each of seven trials to lifestyle intervention or standard care. Individual participant data were combined and analyzed using generalized linear mixed models with trial entered as a random effect. The 12-month assessment was completed by 83% (959/1150) of women and 84% (961/1150) of infants.

**Results:**

Compared with standard care, lifestyle intervention reduced postpartum weight retention (2.2 ± 7.0 vs. 0.7 ± 6.2 kg, respectively; difference of −1.6 kg (95% CI −2.5, −0.7; *p* = 0.0003); the intervention effect was mediated by reduction in excess GWG, which explained 22% of the effect on postpartum weight retention. Lifestyle intervention also significantly increased the odds (OR = 1.68 (95% CI, 1.26, 2.24)) and percentage of mothers (48.2% vs. 36.2%) at or below baseline weight at 12 months postpartum (yes/no) compared with standard care. There was no statistically significant treatment group effect on infant anthropometric outcomes at 12 months.

**Conclusions:**

Compared with standard care, lifestyle interventions initiated in pregnancy and focused on healthy eating, increased physical activity, and other behavioral strategies resulted in significantly less weight retention but similar infant anthropometric outcomes at 12 months postpartum in a large, diverse US population of women with overweight and obesity.

## Introduction

Excess gestational weight gain (GWG) is associated with several adverse pregnancy outcomes and is a well-established risk factor for high maternal postpartum weight retention [1, 2] and greater offspring BMI through childhood and adolescence [3, 4]. Several studies have tested the efficacy of lifestyle interventions to reduce excess GWG [5]. Although effective interventions in women with overweight and obesity had been lacking, the LIFE-Moms (Lifestyle Interventions for Expectant Moms) consortium of seven clinical trials found that comprehensive lifestyle interventions targeting dietary intake, physical activity, and other behavioral factors reduced excess GWG in geographically and socio-demographically diverse populations of women with overweight and obesity [6].

In addition to reducing excess GWG, a major impetus for developing prenatal lifestyle interventions was to reduce postpartum weight retention in women with overweight and obesity. The 12-month postpartum period is an especially important phase for evaluation because weight retention during this time predicts long-term (up to 15 year) subsequent weight gain in women [7, 8]. In children, excessive weight gain during the first 12 months of life is a risk factor for later childhood and adulthood obesity [9]. Understanding the effects of prenatal interventions on 12-month maternal and child weight outcomes could inform future obesity prevention efforts. Other prenatal intervention trials have found no significant group differences in postpartum weight retention [10–15] or infant anthropometric outcomes at months 6–12 [13, 15–17] or 18 and beyond [18, 19]; however, several of these interventions [10, 12, 13, 19] did not reduce GWG, which is the strongest predictor of postpartum weight retention [20]. It is possible that greater reductions in GWG than those seen to date are needed to reduce subsequent postpartum weight retention in women with overweight and obesity. Few studies have examined 12-month postpartum outcomes of effective prenatal lifestyle interventions [10, 12, 15, 21].

The purpose of the current study was to evaluate the effects of the LIFE-Moms prenatal lifestyle interventions on maternal and child anthropometric outcomes through 12 months postpartum. We hypothesized that the LIFE-Moms lifestyle interventions would reduce maternal weight retention (defined as the difference in weight measured early in pregnancy and 12 months postpartum) and increase the percent of women at or below their early pregnancy weight by 12 months postpartum. We also hypothesized that these lifestyle interventions would reduce child weight-for-length *z* scores and adiposity as measured by skinfolds from birth through 12 months of age.

## Materials and methods

### Design

The LIFE-Moms consortium (NCT01545934, NCT01616147, NCT01771133, NCT01631747, NCT01768793, NCT01610752, NCT01812694) was a collaboration of seven clinical centers, a research coordinating unit, and the National Institutes of Health. As previously described [22], each clinical center conducted a separate randomized clinical trial to test strategies intended to reduce GWG in populations with different racial/ethnic and socioeconomic characteristics. Selected eligibility criteria, measures, and procedures were standardized across all trials to enable optimal data pooling and meta-analysis.

### Participants

Institutional review boards for each site and the LIFE-Moms Data and Safety Monitoring Board approved and monitored the conduct of the trials and consortium activities. Study participants provided written informed consent prior to participation. Across the seven trials, recruitment occurred primarily through obstetrician-gynecologist offices between November 2012 and December 2015. As described previously [22], the seven trials recruited participants in the following geographical regions and academic settings: (1) Healthy Beginnings; San Luis Obispo, California; California Polytechnic State University [Cal Poly]; and, Providence, Rhode Island; Brown University; (2) Lifestyle Intervention for Two (LIFT); New York, New York; Columbia University; (3) Pregnancy and EARly Life improvement Study (PEARLS); San Juan, Puerto Rico; University of Puerto Rico; (4) Maternal-Offspring Metabolics: Family Intervention Trial (MOMFIT); Chicago, Illinois; Northwestern University; (5) PreGO; St Louis, Missouri; Washington University; (6) Expecting Success; Baton Rouge, Louisiana; Pennington Biomedical Research Center, and; (7) Phoenix LIFE-Moms; Phoenix, Arizona; NIDDK/Phoenix Indian Medical Center. Common eligibility criteria across the trials included gestational age between 9 and 16 weeks assessed by ultrasound, body mass index ≥ 25 kg/m^2^ based on study entry measured weight and height, and singleton pregnancy. Women were excluded for maternal age < 18 years, diagnosis of diabetes prior to pregnancy or study assessed HbA1c ≥ 6.5% prior to randomization, history of three or more consecutive first trimester miscarriages, current use of exclusionary medications, contraindications to aerobic exercise in pregnancy and additional exclusion criteria [22]. Recruitment in three trials was stopped early (PEARLS, Expecting Success, and Phoenix LIFE-Moms) by the NIH based on the recommendation of the LIFE-Moms Data and Safety Monitoring Board owing to the projected unlikelihood of accruing the target sample size within the time period allowed.

### Standard care

Within each trial, eligible participants were randomly assigned to the local site intervention or to a comparison standard care group. Participants in the standard care group received all aspects of usual care offered by their prenatal care providers. Six of the seven trials supplemented standard care with additional educational content about pregnancy and postpartum health; these materials were provided throughout the study mainly for retention purposes.

### Interventions

By design, the interventions varied across the seven trials. However, all interventions included dietary, physical activity, and behavioral strategies (e.g., self-monitoring, stimulus control, problem solving). Unique intervention elements included the use of meal replacements (Cal Poly/Brown) [23], a modified Diabetes Prevention Program intervention (Columbia; [24] NIDDK/Phoenix), the provision of whole grain, vegetable oil/spread and water (University of Puerto Rico) [25], the DASH diet (Northwestern) [26], a parent educator intervention (Washington University) [27], and a smartphone-based intervention (Pennington) [22]. During pregnancy, women were offered ongoing contact with intervention staff that ranged from weekly to monthly visits across the seven trials. Whereas three trials (Cal Poly/Brown, Pennington, and NIDDK/Phoenix) stopped the intervention after delivery, four trials (Columbia, University of Puerto Rico, Northwestern, and Washington University) continued lifestyle intervention into the postpartum period. Among the trials continuing the lifestyle interventions postpartum, the frequency of intervention visits was reduced from that during pregnancy to monthly or less frequent; the goal was to promote postpartum weight loss that would contribute to an overall reduction in weight retained from pre-pregnancy [22]. The trials with postpartum interventions continued to target healthy eating, physical activity, and weight control behaviors, and three of the four trials also added content pertaining to infant nutrition and feeding [22].

### Outcome assessments

Standardized outcome measures were collected at baseline (9–15 weeks gestation), 24–27 weeks gestation, 35–36 weeks gestation, delivery (within 14 days), and 48–56 weeks postpartum by assessors who were masked to randomization. The Research Coordinating Unit centrally collected and processed all standardized measures prospectively. Data were checked for missing and out of range values and inconsistencies both within and across forms. Participants were provided with monetary compensation for completion of assessment visits that varied across the sites ($50–$150).

Demographic and weight history information were obtained at baseline. Race and ethnicity, demographic factors, and childbearing history were assessed by self-report using questionnaires with fixed categories [22]. A stadiometer was used to measure maternal height in duplicate to the ~ 0.1 cm at baseline. At all assessment visits, maternal weight was assessed in duplicate to the ~ 0.1 kg using a calibrated standard digital scale with the participant in lightweight clothing without shoes. Net postpartum weight retention from baseline was defined as the difference between study measured maternal weight at baseline and weight measured at the 12-month postpartum visit. To allow for comparisons with other studies [12, 28], net postpartum weight retention from pre-pregnancy weight was also computed and defined as the difference between maternal self-reported pre-pregnancy weight and study-measured weight at the 12-month postpartum visit. Although self-reported pre-pregnancy weight is subject to bias, a recent systematic review concluded that there were high correlations between measured and self-reported pre-pregnancy weight [29]. Percent weight retention was defined as postpartum weight retention divided by the starting weight (either the maternal weight at baseline or the pre-pregnancy weight) and multiplied by 100.

Excess GWG per week was assessed as a potential intervention mediator and defined based on Institute of Medicine Guidelines for second and third trimester weight gain as > 0.33 kg/week for women with overweight and > 0.27 kg/week for women with obesity. GWG per week during second and third trimester was calculated as the difference between study measured weight at 35–36-weeks gestation and baseline weight and divided by the number of weeks (days/7) between the two visits. In cases where baseline weight was obtained in the first trimester, the weight was carried forward to the beginning of the second trimester (i.e., 13 weeks 6 days gestation) [6]. (Note that analyses without baseline weight carried forward resulted in similar findings). Breastfeeding was assessed by participants’ self-report at the 12-month postpartum visit.

Neonatal and infant weight, length and skinfold thicknesses were measured at birth (within 14 days) and 48–56 weeks postpartum by centrally trained and certified research staff. Weight was measured using a calibrated scale and length was measured using a standardized board. All assessments were performed in duplicate and if the values differed by a specified amount (> 0.1 kg for weight, > 0.5 cm for length, and > 0.5 mm for skinfold thickness), a third measurement was taken. The average of the closest two measurements was used in data analyses. Birth weight was obtained from medical records. For birth length, given improved accuracy using standardized boards and procedures, study-measured length was used if obtained within 3 days of birth (*N* = 666/919; 72%); chart-abstracted length was used if the measured length was obtained beyond 3 days (*N* = 253/919; 28%). Skinfold thickness was measured by trained staff in duplicate using the Harpenden skinfold caliper on right side of the body at the following sites: triceps, subscapular, thigh, and iliac crest. Skinfolds for preterm infants (< 37 weeks 0 days; *n* = 65) were not included in the analyses. Weight-for-length, triceps, and subscapular *z* scores were calculated using the WHO Child Growth Standards for age and sex [30].

### Statistical analyses

All non-pregnant mothers and infants who completed a 1-year study visit were included in the analysis, regardless of whether the visit was performed within (48–56 weeks postpartum) or outside the visit window; sensitivity analyses were performed including only visits completed within the study visit window. We also compared individuals who did and did not complete the 12-month visit. Given that there were no standardized visits between delivery and the 12-month visit, imputation for the 12-month outcomes was not considered.

A one-stage individual patient data meta-analysis was conducted using data from the seven randomized trials. The effect of the intervention on each outcome was analyzed using generalized linear mixed models with intervention as a fixed effect and “trial” entered as a random effect to account for potential differences in the study population. Intervention effect was included in the models as a fixed effect because: (1) the interventions all targeted the same diet, physical activity, and behavioral strategies; (2) the estimated effect sizes reported in the trial protocols were similar; and (3) the goal of LIFE-Moms was to estimate one common effect rather than the mean of a distribution of effects. All participants in the standard care groups were included as one “standard care” group, and all participants in the intervention groups were included as one intervention group. Data from all women were analyzed according to the group to which they were randomly assigned, regardless of whether they adhered to the lifestyle intervention. Outcome and subgroup analyses were pre-specified. In addition, the following covariates were included in each model: Maternal BMI category at baseline (overweight, obese), parity (nulliparous, multiparous), maternal college education, maternal age category at baseline (18–24, 25–29, ≥ 30 years), gestational age at randomization (< 13, ≥ 13 weeks), and infant sex. *z* scores were not adjusted for infant sex because sex was accounted for in the standardization procedure. Infant anthropometrics evaluating change from birth to 12 months postpartum also included the anthropometric measure at birth as a covariate.

Additional analyses examined group × demographic subgroup moderator effects. Subgroups included baseline BMI category (overweight, obese), college education (yes, no), baseline maternal age (18–24, 25–29, ≥ 30 years), nulliparous (yes, no), and gestational age at randomization (< 13, ≥ 13 weeks).

Other analyses explored whether the effect of treatment group was mediated by excess GWG or breastfeeding. Treatment group differences in excess GWG (yes vs. no) and breastfeeding duration were first examined in relation to postpartum weight retention from baseline. If there was a statistically significant treatment group difference with the mediator, and the mediator was associated with the outcome, subsequent analysis examined whether the effect of treatment group on postpartum weight retention was changed after inclusion of the potential mediator in the model. To evaluate the impact of continuing vs. stopping the intervention postpartum, a post-hoc analysis was performed comparing studies that included or excluded a postpartum intervention.

For all outcomes, nominal *p* values of < 0.05 were considered to indicate statistical significance; *p* values have not been adjusted for multiple comparisons. Analyses were performed using SAS version 9.4 (SAS Institute, Cary, NC).

## Results

**Fig. 1:**
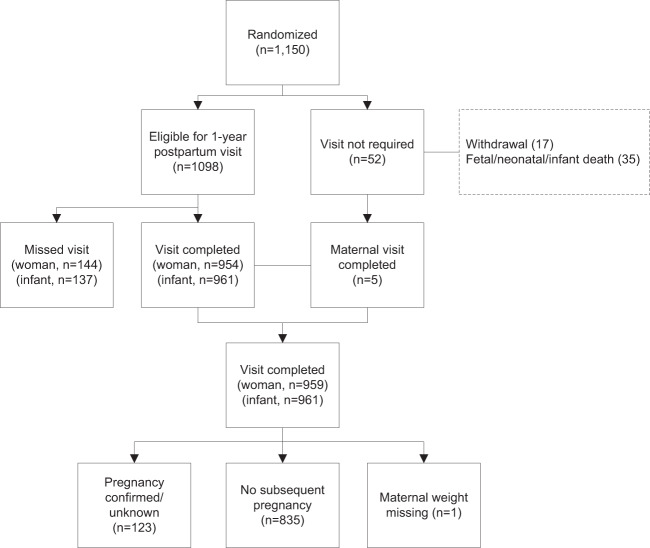
Participant flow in LIFE-Moms

Figure 1 summarizes the participant flow and retention in LIFE-Moms. After excluding formal withdrawals (*N* = 17) and women with fetal/infant losses who did not complete a 12-month follow-up visit (*N* = 30), the 12-month postpartum visit was completed by 959/1103 (87%) of women, and most (763/959; 80%) of these visits were completed within the 48–56-week postpartum window. (Note that five women experienced a fetal/infant loss late in pregnancy (≥ 34 weeks gestation) and completed the 12-month postpartum visit and were included in analyses.) Women with a confirmed or unknown subsequent pregnancy since the LIFE-Moms delivery were excluded from the analyses evaluating postpartum weight retention (*N* = 123). The demographic characteristics did not significantly differ between women and infants who attended vs. did not attend the 12-month postpartum visit. After excluding other fetal/neonatal/infant deaths (*N* = 35) and withdrawals (*N* = 17), the 12-month visit was completed by 961/1098 (88%) of infants. Most (771/961; 80%) visits were completed within the 48–56-week postpartum window.

### Maternal postpartum weight retention

Characteristics of the women were well balanced by randomized groups (Table 1). As shown, 35% of the women were Non-Hispanic Caucasian, 32% were Non-Hispanic African American, and 24% of the women were Hispanic. Also, 43% of women were classified with overweight and 57% with obesity.

**Table 1:** Baseline characteristics of women and infants in LIFE-Moms^a^

	Intervention (*N* = 579)	Standard of care (*N* = 571)
*Maternal*		
Gestational age at randomization (wk)	14.1 [12.7–15.1]	14.1 [12.6–15.3]
Maternal age (yr)	30.4 ± 5.6	30.5 ± 5.7
Weight unadjusted at screening (kg)	82.4 [72.9, 93.4]	83.1 [74.0, 94.8]
BMI unadjusted at screening (kg/m^2^)	30.7 [27.8–34.6]	30.8 [28.2–35.0]
BMI category at screening		
Overweight	253 (43.7%)	242 (42.4%)
Obese	326 (56.3%)	329 (57.6%)
Pre-pregnancy self-reported weight (kg)	80.1 [71.7, 91.6]	80.7 [72.1, 92.5]
Pre-pregnancy BMI category (kg/m^2^)^b^		
Normal	37/576 (6.4%)	32/565 (5.7%)
Overweight	256/576 (44.4%)	238/565 (42.1%)
Obese	283/576 (49.1%)	295/565 (52.2%)
Race/ethnicity		
Non-Hispanic Caucasian	196 (33.9%)	205 (35.9%)
Non-Hispanic African Am.	193 (33.3%)	180 (31.5%)
Hispanic	138 (23.8%)	133 (23.3%)
Other, more than one race	52 (9.0%)	53 (9.3%)
College education	291 (50.4%)	279 (48.9%)
Total family income		
<$25,000	198 (34.6%)	209 (36.8%)
$25,000–$74,999	159 (27.8%)	151 (26.6%)
≥$75,000	215 (37.6%)	208 (36.6%)
Married/living with significant other	435 (75.3%)	440 (77.1%)
Nulliparous	254 (43.9%)	219 (38.4%)
*Child*		
Child sex^c^		
Male	250/567 (44.1%)	289/550 (52.5%)
Female	317/567 (55.9%)	261/550 (47.5%)
Child race/ethnicity^d^		
Non-Hispanic Caucasian	142/467 (30.4%)	137/454 (30.2%)
Non-Hispanic African Am.	147/467 (31.5%)	135/454 (29.7%)
Hispanic	125/467 (26.8%)	127/454 (28.0%)
Other, more than one race	53/467 (11.3%)	55/454 (12.1%)

^a^Data presented as *N* (percent), mean ± standard deviation, or median [inter-quartile range]

^b^Note that these data are based on self-reported pre-pregnancy weight. All women met criteria for overweight or obesity based on the first prenatal, study entry measurement

^c^All group differences are non-significant except for fetal sex with *p* = 0.005

^d^Available only on infants that completed the questionnaire for the 1-year postpartum visit (*N* = 921)

**Fig. 2:**
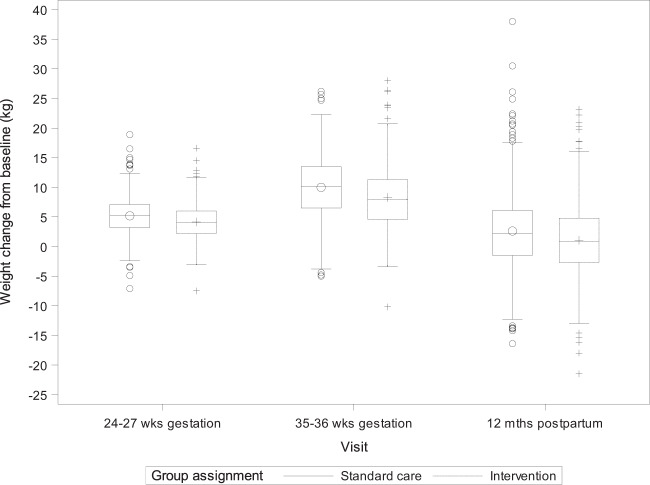
Weight change from baseline (i.e., early pregnancy) across visits. The dashed lines denoting the intervention are not appearing on this figure as they should; dahsed lines were included in the figure that was uploaded

**Fig. 3:**
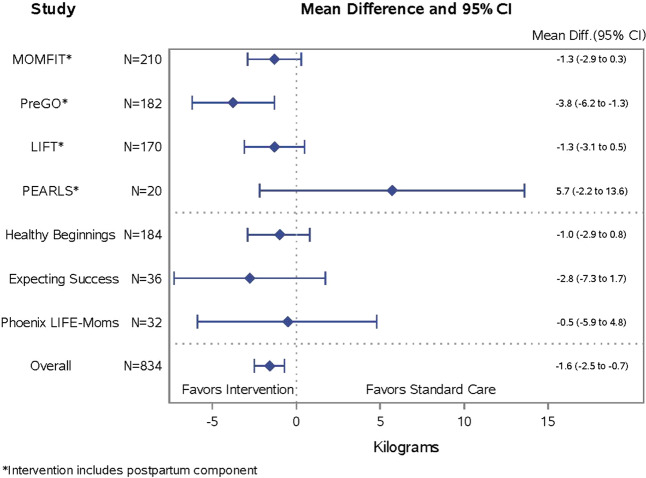
Forest plot of postpartum weight retention by trial

Weight change variables for the postpartum period are summarized in Table 2 and Figs. 2, 3. Lifestyle intervention significantly (*p* < 0.001) reduced postpartum weight retention from “baseline” weight (i.e., early pregnancy weight) relative to standard care, and the adjusted mean group difference in postpartum weight retention was −1.6 kg (95% CI, −2.5, −0.7). Similarly, lifestyle intervention relative to standard care significantly increased the odds (OR = 1.68 (95% CI, 1.26, 2.24)) and percentage of mothers (48.2 vs. 36.2%) achieving baseline weight or below by 12 months postpartum (yes/no). Similar results were obtained examining intervention effects on postpartum weight retention calculated from self-reported pre-pregnancy weight (Table 2) or when the analyses were restricted to women who had their 12-month postpartum visit within the window (48–56 weeks postpartum, data not shown).

**Table 2:** Effect of LIFE-Moms interventions on maternal postpartum weight retention at 12 months

	Intervention (*n* = 423)	Standard care (*n* = 412)	Adjusted mean difference (95% CI)^a^	Adjusted odds ratio^a^
Postpartum weight retention at 12 months relative to baseline (i.e., early pregnancy) weight				
Net weight retention, kg ± SD	0.7 ± 6.2	2.2 ± 7.0	−1.6 (−2.5, −0.7)	
Percent weight retention, % ± SD^b^	0.8 ± 7.6	2.5 ± 8.3	−1.8 (−2.8, −0.7)	
At or below baseline weight, no. (%)	204 (48.2%)	149 (36.2%)		1.68 (1.26, 2.24
>0–< 5% of baseline weight, no. (%)	99 (23.4%)	129 (31.3%)		
5–< 10% of baseline weight, no. (%)	74 (17.5%)	66 (16.0%)		
≥10% of baseline weight, no. (%)	46 (10.9%)	68 (16.5%)		
Postpartum weight retention at 12 months relative to pre-pregnancy weight^c^				
Net weight retention, kg ± SD	2.4 ± 6.5	4.1 ± 7.4	−1.7 (−2.6, −0.7)	
Percent weight retention, % ± SD^b^	3.2 ± 8.1	5.0 ± 9.3	−1.9 (−3.1, −0.8)	
At or below pre-pregnancy weight, no. (%)	148/421 (35.2%)	112/407 (27.5%)		1.46 (1.08, 1.98)
>0–< % of pre-pregnancy weight, no. (%)	116/421 (27.6%)	108/407 (26.5%)		
5–< 10% of pre-pregnancy weight, no. (%)	73/421 (17.3%)	88/407 (21.6%)		
≥ 10% of pre-pregnancy weight, no. (%)	84/421 (20.0%)	99/407 (24.3%)		

^a^Parameter estimates with adjustments for gestational age at randomization, baseline BMI; parity, college education, maternal age, infant sex, and trial as a random effect

^b^Percent weight retention was defined as postpartum weight retention divided by the starting weight used (either the maternal weight at baseline or the pre-pregnancy weight) and multiplied by 100

^c^Based on self-reported weight; seven women were missing pre-pregnancy weight (two intervention, five standard care)

There were no significant group effect moderators (i.e., BMI category, education, maternal age, parity, gestational age at randomization, infant sex) in relation to 12-month postpartum weight retention (i.e., weight retained from baseline/early pregnancy). In exploring potential intervention mediators, the effect of treatment group on postpartum weight retention from baseline was significantly (*p* = 0.005) reduced after GWG was entered into the model. Specifically, the adjusted between-group difference in weight retention was reduced from −1.6 (−2.5, −0.7) to −1.2 kg (−2.1, −0.4) after excess GWG was entered into the model, explaining 22% of the group effect on 12-month postpartum weight retention. Breastfeeding did not appear to mediate intervention effects on postpartum weight retention.

In post-hoc analyses among women with no subsequent pregnancy (reported at the 12-month visit), the 423 women in the intervention group were further classified into prenatal and postnatal intervention (293/835, 35.1%) and prenatal only intervention (130/835, 15.6%). Women randomized to the combined prenatal and postpartum interventions had the least postpartum weight retention (mean ± standard deviation, 0.5 ± 6.4 kg), followed by women randomized to the prenatal only intervention (1.1 ± 5.7 kg), and then standard care (2.2 ± 7.0 kg). The adjusted mean difference relative to standard care was −1.9 (−2.8, −0.9 kg) for prenatal and postnatal intervention and −1.1 (−2.4, 0.3 kg) for prenatal intervention only. Figure 3 illustrates the effect of treatment group on postpartum weight retention among the trials that included prenatal and postpartum intervention followed by the trials with prenatal intervention only. We also explored GWG was a mediator of the effects of prenatal only and combined prenatal and postpartum interventions on postpartum weight retention. GWG explained 30% of the group effect for those with a prenatal intervention only, and 21% of the group effect for those with a prenatal and postnatal intervention, when compared with standard care.

### Infant outcomes

As shown in Table 1, 30% of the infants were Non-Hispanic Caucasian, 31% were Non-Hispanic African American, and 27% were Hispanic. There were more male than female infants born in standard care than intervention groups; this variable was adjusted for in all analyses.

Changes in infant anthropometrics and skinfolds measures are summarized in Table 3. The intervention had no statistically significant effect on changes in infant weight-for-length, triceps skinfold for age *z* scores, subscapular skinfold for age *z* scores, or thigh and iliac crest skinfold measurements between birth and 12 months of age in analyses that adjusted for maternal characteristics (i.e., baseline BMI, parity, college, age, gestational age at randomization) and infant sex. Similar results were seen when the analyses were restricted to infants who had their 12-month postpartum visit within the window (48–56 weeks postpartum, data not shown).

**Table 3:** Effect of LIFE-Moms interventions on offspring anthropometrics at 12 months

	No of infants	Intervention	Standard care	Adjusted mean difference (95% CI)
Weight for length, *z* score	957	0.33 ± 1.04	0.35 ± 0.92	−0.01 (−0.13, 0.11)
Weight for length *z* score at birth	919	−0.18 ± 1.28	−0.28 ± 1.29	0.13 (−0.04, 0.29)
Change from birth to 12 months of age	916	0.55 ± 1.55	0.61 ± 1.52	0.03 (−0.09, 0.15)
Triceps skinfold for age, *z* score	953	0.62 ± 1.27	0.63 ± 1.29	−0.03 (−0.19, 0.13)
Triceps SF, cm at birth	822	4.93 ± 1.16	4.99 ± 1.28	−0.07 (−0.23, 0.09)
Triceps SF, cm at 12 months of age	953	9.43 ± 2.42	9.44 ± 2.47	−0.10 (−0.40, 0.21)
Change from birth to 12 months of age	814	4.55 ± 2.51	4.50 ± 2.56	−0.09 (−0.41, 0.23)
Subscapular skinfold for age, *z* score	953	0.46 ± 1.27	0.46 ± 1.17	0.01 (−0.15, 0.16)
Subscapular SF, cm at birth	822	4.73 ± 1.15	4.70 ± 1.18	0.02 (−0.13, 0.18)
Subscapular SF, cm at 12 months of age	953	7.34 ± 1.87	7.27 ± 1.76	0.04 (−0.19, 0.27)
Change from birth to 12 months of age	814	2.68 ± 2.03	2.58 ± 1.89	0.08 (−0.17, 0.32)
Thigh skinfold	952	16.30 ± 3.93	16.43 ± 3.66	−0.17 (−0.65, 0.31)
Thigh SF, cm at birth	822	6.48 ± 1.63	6.38 ± 1.61	0.05 (−0.17, 0.26)
Change from birth to 12 months of age	813	9.92 ± 4.27	10.07 ± 3.86	−0.11 (−0.63, 0.40)
Iliac crest skinfold	952	8.74 ± 3.17	8.83 ± 2.98	−0.05 (−0.42, 0.33)
Iliac crest SF, cm at birth	822	4.43 ± 1.27	4.46 ± 1.31	−0.03 (−0.20, 0.14)
Change from birth to 12 months of age	813	4.42 ± 3.30	4.39 ± 3.02	−0.03 (−0.43, 0.37)

*SF* skinfold. Parameter estimates with adjustments for gestational age at randomization, baseline BMI; parity, college education, maternal age, and trial as random effect. Variables other than *z* scores also included infant sex, and variables evaluating change from birth included the measurement at birth

In both groups over time, weight-for-length and skinfold *z* scores increased (Table 3). Prevalence of overweight (i.e., weight-for-length *z* score > 2.0 SD) was low in the intervention and standard care groups at birth (2.2% and 2.8%, respectively) and 12 months of age (4.9% and 3.6%, respectively). Prevalence of underweight (i.e., weight-for-length *z* score <2.0 SD) was also low in intervention and standard groups at birth (8.3% and 9.6%, respectively) and 12 months (1.6% and 0.4%, respectively). We explored whether infant weight-for-length and skinfold z scores differed by prenatal intervention only, prenatal and postnatal intervention, and standard care groups, and no statistically significant differences emerged (data not shown).

## Discussion

Using a prospective, individual participant data meta-analysis, this study combined data from seven clinical trials that used the same core measurement protocol but tested different prenatal lifestyle interventions. We previously reported that the lifestyle interventions reduced excess GWG [6]. This study demonstrated that the interventions had a lasting effect on maternal weight through the postpartum period. The lifestyle interventions reduced 12-month postpartum weight retention by 1.6 kg and increased by ~70% the odds that women would achieve either their baseline (i.e., early pregnancy) weight or a lower weight by 12 months postpartum. The intervention effect did not differ across selected demographic strata.

Findings from prior studies testing the long-term effects of prenatal interventions in women with overweight and obesity have been mixed. Five studies [10–14] found no significant effects of prenatal interventions on 12-month postpartum weight retention, but four of these [10, 12, 13, 19] did not reduce excess GWG in women with obesity. In the current study, the 1.6 kg lower GWG [6] and the 1.6 kg reduction in weight retention at 1 year should be considered within the context of the target population of young adult women with overweight/obesity preconception. Weight gains of 0.8–1 kg per year in young adults increase cardiovascular disease risk [31]. Even modest (≥ 1 kg) postpartum weight retention is linked to increased risk of later weight gain and development of obesity and diabetes in women [8].

The intervention-related prevention of excess GWG explained 22% of the effect on 12-month postpartum weight retention. The LIFE-Moms findings align with prior research showing that GWG is the strongest predictor of postpartum weight retention [20]. Breastfeeding did not appear to mediate the effects of the interventions, but this analysis may be underpowered, as it was only targeted in three of the seven LIFE-Moms trials [22]. The remaining unexplained variance suggested that other factors played a role in the observed group effect on postpartum weight retention.

Findings from this study suggested that continuing interventions postpartum may positively benefit postpartum weight retention. Prenatal interventions that continued during the postpartum year resulted in the least postpartum weight retained (0.5 kg), followed by prenatal only intervention (1.1 kg), and standard care (2.2 kg). Also, the trial that appeared to have the strongest effect on reducing postpartum weight retention (PreGO trial; Fig. 3) also had an intervention that continued postpartum. Also, in studies that continued interventions postpartum (vs. prenatal intervention only), the positive effects on postpartum weight retention were less explained by variance in GWG alone (21% vs 30%, respectively), suggesting that continuing intervention postpartum might have provided additional avenues–beyond GWG- to affect postpartum weight retention. The postpartum interventions in the current study were of relatively low intensity (monthly or less-frequent contact) compared with other postpartum interventions, yet appeared to have a significant benefit [12, 20, 32–34]. Initiating lifestyle interventions during pregnancy may offset the need for (and cost of) intensive face-to-face interventions during the postpartum period that can be especially challenging for women to follow owing to time constraints, feeding and childcare demands, stress, fatigue, and reduced social support [35–37]. It is also possible that more-intensive postpartum interventions—if geared to the new mom’s schedule and using more convenient electronic methods—could augment effects on reducing postpartum weight retention [32]. Published postpartum interventions have reported mixed results [12, 20], but significant weight loss has been reported in behavioral interventions delivered via the internet [32], mail [33], and phone [34]. As LIFE-Moms was designed as a meta-analysis that aggregated across the trials, these post hoc analyses comparing interventions that stopped vs. continued during the postpartum period should be interpreted with caution.

The interventions had no significant effect on infant weight gain and adiposity changes from birth through 12 months. Few clinical trials have tested the long-term effects of successful prenatal interventions on offspring outcomes. Studies that did not reduce excess GWG have not found significant group differences on infant anthropometrics [13, 17, 18]. Studies that successfully reduced excess GWG have shown mixed findings. A UK-based trial found that a low-glycemic index diet intervention that reduced excess GWG also reduced child subscapular but not triceps skinfold thickness at 6 months [16]. A Finnish trial with follow-up beyond 1 year found positive effects of a prenatal probiotic intervention in reducing child BMI at 4 years of age [38]. On the other hand, a US trial, similar to the current trials, found that a lifestyle intervention that reduced excess GWG in women with obesity had no significant effect on child weight-for-length *z* score or skinfolds at 1 year of age [11]. Other prenatal intervention trials that reduced excess GWG [19, 39] did not observe any significant effects on infant anthropometrics at 6 months [39] or 3 years [19]. It is possible that prenatal interventions that begin late in the first trimester do not influence neonatal or early childhood growth trajectories; earlier interventions may be needed to affect early metabolic imprinting in offspring [40]. It is also possible that prenatal interventions need to produce greater reductions in GWG or will have effects on offspring that do not emerge until the later childhood years, as suggested by some observational studies [41–43].

The LIFE-Moms study has several strengths and some limitations. The LIFE-Moms study is the first prospective individual participant meta-analysis to include a priori hypotheses and standardized core measures while testing a variety of lifestyle intervention approaches during pregnancy. Participants were from diverse sociodemographic and geographic backgrounds, increasing potential generalizability. Despite these strengths, some clinical sites were comprised of only a single race/ethnic group, limiting analysis of this variable as a group moderator across all the trials. Although anthropometrics were measured by centrally trained staff, using standardized, repeated measures of skinfolds and circumferences, the study did not include measures of dual-energy X-ray absorptiometry that could be more sensitive to changes over time, particularly in the infants. Also, the LIFE-Moms study lacked an intermediate postpartum measure (e.g., at 6 months) to inform patterns over time. Finally, statistical models adjusted for several potential confounding variables related to postpartum weight retention but age at menarche, time from menarche to pregnancy, and other potential confounding variables were not included [44].

In conclusion, behavioral lifestyle interventions that were initiated early in pregnancy and focused on healthy dietary, physical activity, and behavioral strategies resulted in significantly less 12-month postpartum weight retention compared with standard care. This beneficial effect was seen across diverse interventions and in a large, racial, and socioeconomic diverse US population.
